# Preclinical Models of Oropouche Virus Infection and Disease

**DOI:** 10.3390/pathogens14121272

**Published:** 2025-12-11

**Authors:** Daniel Morley, Emma Kennedy, Stuart Dowall

**Affiliations:** UK Health Security Agency, Porton Down, Wiltshire SP4 0JG, UK; daniel.morley@ukhsa.gov.uk (D.M.); emma.kennedy@ukhsa.gov.uk (E.K.)

**Keywords:** oropouche, arbovirus, preclinical, mouse, hamster, non-human primate, model

## Abstract

Oropouche virus (OROV) is an emerging and underreported arbovirus with dengue-like symptoms confounding diagnosis. OROV is also neuroinvasive, with a small number of cases presenting severe neurological symptoms. There have been recently reported deaths from confirmed cases of OROV and reported instances of vertical transmission from mother to foetus, with confirmed cases in Brazil and a congenital anomaly, reportedly as a consequence of OROV infection in Cuba, with further cases under investigation. Whilst cases of OROV infection occur mainly in South America, many cases have been imported elsewhere, including the United States and Europe. Despite the emerging threat to public health, animal modelling to study OROV pathogenicity and immunity and to evaluate therapeutic candidates remains limited. For this review, we carried out a literature search through major research databases (PubMed and Scopus) up to September 2025 to capture the extent of in vivo model development for this pathogen. We identified only 17 relevant primary research articles within these criteria which detailed hamster, mouse and non-human primate (NHP) models. Here, we discuss the extent of in vivo model development for OROV. In summary, small and large animal models need to be assessed with recent clinical isolates and reassortants, asymptomatic disease presentation in the NHP model requires further study and the hamster model shows potential for use in pathogenicity and vaccine or antiviral efficacy studies. We also compile relevant metadata and discuss the need for an animal model that more closely resembles human disease.

## 1. Introduction

First discovered in Vega de Oropouche, Trinidad in 1955 [1], OROV is endemic to South America, where it causes sporadic outbreaks, primarily in Brazil, with over 500,000 cases reported [2]. OROV is a zoonotic arbovirus that transmits through sylvatic and urban cycles, its main vector being the biting midge *Culicoides paraensis*, though many other regional vectors with lower transmission efficiency, such as the *Aedes aegypti* and *Culex quinquefasciatus* mosquitos, have been identified [3,4,5]. OROV amplifies in humans during its urban cycle and outbreaks occur primarily in the rainy season or at the beginning of the dry season, when the populations of OROV vectors peak [6]. No horizontal transmission between humans has been reported. Multiple sylvatic reservoirs have been confirmed, including sloths, non-human primates and wild birds [7]. Given that there are multiple susceptible avian families with members distributed across North and South America with north/south migration patterns, there is risk of introduction events in Latin America and the United States [8,9,10]. There are also reported instances of vertical transmission from mother to foetus [11]. The possibility of sexual transmission has also been hypothesised due to a case in Italy in 2024, where prolonged viral shedding was detected by RT-PCR in the semen of a patient diagnosed with Oropouche fever, though this has not been confirmed [12].

Dozens of outbreaks have occurred since 1955, with the majority located in northern Brazil and several in Panama and, since the year 2000, outbreaks have also been confirmed in Argentina, Bolivia, Colombia, Cuba, Ecuador, Guyana and Peru [13]. Imported cases to Europe have also been reported, with Germany, Italy and Spain reporting their first ever cases of OROV infection in 2024 [14].

OROV is a negative-sense single-stranded RNA virus of the genus Orthobunyavirus in the Peribunyaviridae family. As with all bunyaviruses, OROV has a segmented tri-partite genome consisting of a 1.0 kb small (S) segment, 4.5 kb medium (M) segment, and a 6.9 kb large (L) segment. Each segment is flanked by complementary 3′ and 5′ UTRs which circularise the genome segments and act as promoters [15]. The L segment encodes a 259 kDa RNA-dependent RNA polymerase (RdRp). The M segment encodes the precursor polyprotein Gn-NSm-Gc, which is post-translationally cleaved by host proteases into the surface glycoproteins Gc (28 kDa) and Gn (107 kDa), as well as non-structural proteins NSm (27 kDa), which was shown to enable efficient infection and transmission in the mosquito vector [16]. The S segment encodes both the nucleocapsid protein N (26 kDa) and another non-structural protein NSs (11 kDa) from an overlapping reading frame via leaky ribosomal scanning of the AUG start codon [15].

Four genotypes with different geographical distributions have been identified for OROV, based on sequence variation in sections of the M and S segments encoding the Gn and N proteins, respectively [17]. Notably, due to its segmented genome, OROV is susceptible to reassortment in the event of co-infection. Three OROV M segment reassortants have currently been identified—Iquitos virus (IQTV), Madre de Dios virus (MDDV) and Perdões virus (PERDV) [18,19,20]—and the possibility broader reassortment patterns within the Bunyaviridae family has been considered [20].

There are many gaps in what is currently known about OROV pathogenesis in humans. Onset of symptoms typically occurs after a 3- to 8-day incubation period following transmission via bite. The severity of symptoms varies, with cases resulting from infection by emerging strains able to cause more severe disease [12,21,22]. Symptoms typically persist for 2 to 7 days and include fever, which is reported to occur in nearly all cases; chills, nausea and vomiting are also commonly reported [23]. Haemorrhagic symptoms, reported in approximately 15% of cases, include spontaneous bleeding, typically gingival, subdermal or nasal [23]. The most frequently reported neurological symptoms are headache, myalgia and eye pain [24], and meningitis and meningoencephalitis are reported at much lower rates [23]. Recurrence of mild symptoms reportedly occurs in approximately 60% of cases [19]. Fatalities from cases with Oropouche fever are low and have only recently begun to be reported and cases with severe neurological symptoms remain rare [25]. Case reports show OROV replicates to high titres in the bloodstream, with some cases showing that viraemia persists long after symptoms clear [12,26]. Viral load can also persist in urine and semen. Systemic spread to the liver and kidneys was observed in fatal cases; additional lung damage was also noted in the absence of detectable virus [21]. CNS spread is hypothesised to occur via a ‘trojan horse’ mechanism where OROV bypasses the blood–brain barrier inside peripheral blood monocytes [27]; this is thought to induce an inflammatory response based on TNF-α levels detected in human brain slice cultures following infection by OROV [28].

No antivirals are currently licenced for the treatment of OROV infection, though preliminary research into the efficacy of several antivirals shows promise; these are listed in Table 1. Ribavirin shows in vitro inhibition of OROV at a high concentration [29], but it has also been assessed in mice and was found to be unable to limit viral replication in CNS tissues or reduce the lethality of OROV infection [30]. 4′-fluorouridine has also been shown to be effective in vitro, at much lower concentrations than ribavirin, and conferred full protection from OROV infection in immune-deficient mice when treated with a 10 mg/kg dose [29].

Other candidate antivirals assessed in vitro include acridones, which were shown to inhibit viral replication at multiple steps with high efficiency by interfering with the cap snatching mechanism of the OROV RdRp and binding dsRNA intermediates [31]. Quercetin hydrate, a flavonoid, has been found to bind OROV Gc with high efficiency at low concentrations [32] and was also found to reduce reactive oxygen species (ROS) levels in RNA virus-infected tissues, potentially reducing tissue damage [33]. The nucleoside analogue Favipiravir acts as a chain terminator in the replication of a broad range of RNA viruses and its efficacy as an OROV antiviral has also been assessed [34,35].

**Table 1:** Antivirals that have been assessed for activity against OROV.

Publication	Compound	Class	Mechanism of Action	Evidence Level
Westover et al., 2025 [29]	4′-Fluorouridine	Nucleoside analogue (uridine)	RdRp inhibitor	in vitro, in vivo
Westover et al., 2025 [29]	Ribavirin	Nucleoside analogue (guanosine)	RdRp inhibitor	in vitro
Westover et al., 2025 [29]	Favipiravir	Nucleoside analogue (guanosine)	RdRp inhibitor	in vitro
Saivish et al., 2024 [31]	Acridones	Small molecule	Possible intercalation of dsRNA intermediate during viral replication	in vitro
de Lima Menezes et al., 2023 [32]	Quercetin hydrate (Flavinoid)	Small molecule	Proposed interactions with OROV Gc protein	in vitro

Progress has also been made with the development of an attenuated vaccine strain, with recombinant OROV lacking the NSm and NSs proteins [36]. A chimeric VSV expressing OROV Gc protein was also shown to be immunogenic in BALB/c mice [37]. Preclinical models will be critical for the further assessment of these therapies, alongside ongoing vaccine development work.

The lack of robust animal models has been identified as a barrier to improving our understanding of OROV pathogenesis and developing countermeasures [35,38,39,40]. Animal models are necessary for the investigation of disease pathology and transmission, and for evaluation of the safety and efficacy of candidate therapeutics against OROV and its reassortant strains. Hamsters, mice and non-human primates (NHPs) have seen use in modelling OROV disease and evaluating potential therapeutics. The extent of research undertaken with these models is described below.

## 2. Methods

**Figure 1:**
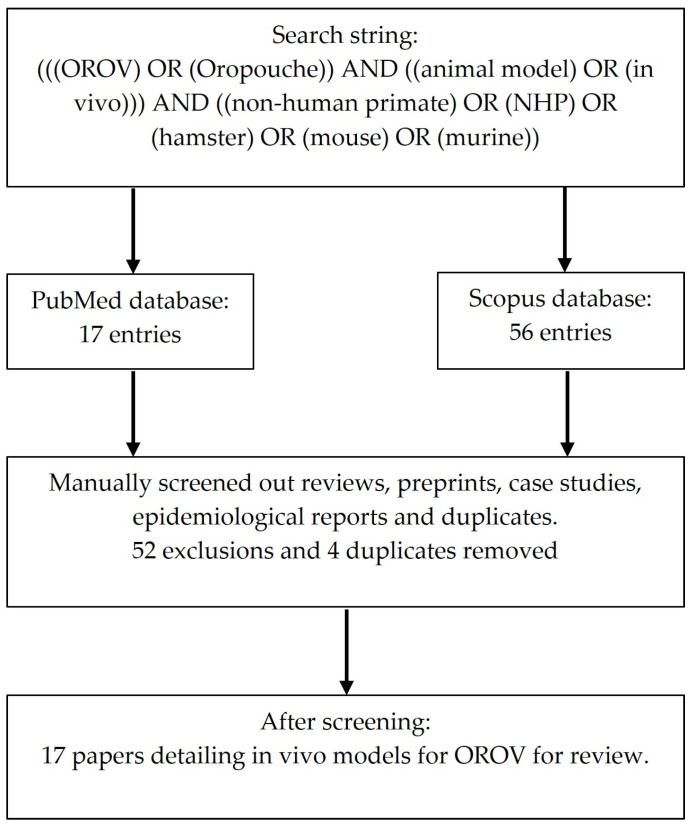
Flow chart of search methodology.

We carried out a literature search in PubMed and Scopus from 1955 up to September 2025. Searches were completed in English with the following search string containing relevant keywords. The search methodology is detailed in Figure 1. From this, we identified primary research articles detailing in vivo models in which animals were challenged with OROV. We collate model metadata and provide a narrative review of model development.

## 3. Models

### 3.1. Hamster Models

In the 1970s and 1980s, a series of vector transmission models were established in Syrian hamsters and experimentally confirmed the biting midge *C. paraensis* and the mosquito *C. quinquefasciatus* as vectors [3,5,41]. These papers, as well as a pathogenicity model in Syrian hamsters, are included in Table 2.

A 2011 paper by Rodrigues et al. demonstrates the utility of Syrian hamsters as a pathogenicity model for OROV, showing viraemia and systemic spread to the brain and liver as well as clinical signs of disease [42]. Initial dose-ranging using BeAn19991 OROV identified the LD_50_ as equivalent to 10^5.6^ TCID_50_/mL. Hamsters were subsequently administered with a 4 LD_50_ subcutaneous challenge of OROV, with over half displaying severe clinical signs after 3 dpi, including reduced feeding and grooming, weight loss, signs of fever, stumbling and hind limb paralysis. Viral titres obtained from blood, brain and liver peaked at 10^6^ TCID_50_/mL by 3 dpi and persisted until the end of study at 11 dpi. Fever spikes were also observed for several hamsters between 4 and 8 dpi [42].

**Table 2:** Overview of OROV hamster models.

Publication	Virus		Animals			Study Design				
	Strain	Passage History	Species	Age	Sex	Challenge Dose	Route of Infection	Inoculation Site	Group *n*	Primary Endpoints
Pinheiro et al., 1982 [3]	BeAn19991	Mouse i.c passage	Syrian hamster	4-week-old	Mixed	5.2–7.3 log_10_ SMLD_50_ *^1^	Midge vector	Abdomen	12–15	Survival, weight loss, transmission confirmed by cell culture, seroconversion to confirm infection
Hoch et al., 1987 [5]	BeAn19991	Mouse i.c passage	Syrian hamster	3-week-old	Mixed	9.7–9.9 log_10_ SMLD_50_ *^1^	Mosquito vector	Abdomen	27–33	Survival, weight loss, transmission confirmed by cell culture, seroconversion to confirm infection
Rodrigues et al., 2011 [42]	BeAn19991	Mouse i.c passage	Syrian hamster	3-week-old	Mixed	10^5.6^ TCID_50_ *^1^	Subcutaneous	Hind leg	13	Survival, weight loss, temperature, clinical scoring, histopathology, viral load

*^1^ Units are as detailed in published work and were not converted due to incompatible assay principles.

### 3.2. Immune-Competent Mouse Models

An overview of immune-competent mouse models used in OROV research is shown in Table 3.

A 2006 study showed ribavirin had no inhibitory effect in neonatal mice challenged with a 10 LD_50_ intraperitoneal dose of OROV BeAn19991. Mice treated 24 h before challenge showed no increased survival rate and no reduction in plaques from when titrating brain homogenates [30].

In 2012, newborn wildtype BALB/c mice were challenged with 10^6.25^ TCID_50_ BeAn19991 OROV via the subdermal route into the dorsal lumbar. A total of 85% of infected mice died after 5 dpi, presenting with weight loss and paralysis. OROV showed strong neurotropism, with virus only being detected in CNS tissues, although inflammation was also observed in the spleen [43]. A follow-up paper in 2014 sought to better characterise the spread of OROV in the mouse central nervous system. The same strain, dose and challenge route were used in newborns. Tissue sections were taken along the length of the spinal cord and in each region of the brain at 3, 4 and 6 dpi. Asymptomatic mice tested positive for OROV in the brainstem by IHC and symptomatic mice showed more severe CNS spread and inflammation. It was hypothesised that CNS spread may have occurred by a non-neuronal route in symptomatic mice, supported by the observation of increased blood–brain barrier permeability (by Evans blue staining) in those animals [44].

A mouse model was used by Stubbs et al. to assess the efficacy of several vesicular stomatitis virus (VSV) constructs expressing OROV glycoproteins Gc and Gn. Groups of six adult male C57BL/6 mice were treated with a prime-boost regimen of VSV-OROV administered intramuscularly. One week after a booster immunisation, mice were challenged with 10^6^ TCID_50_ ORV BeAn19991 via the subcutaneous route into the dorsal lumbar. Vaccinated groups experienced reduced clinical signs of disease and reduced weight loss and saw no post-challenge temperature spike seen in unvaccinated mice. Reduced viral load was observed in CNS tissues, as determined by RT qPCR. VSV-OROV also induced high neutralising antibody titres [37].

OROV pathogenesis was further characterised in 3-week-old BALB/c mice in a 2023 paper by da Silva Menegatto et al. through the identification of virus and oxidative stress biomarkers in the spleen and liver. Mice were challenged subcutaneously with OROV BeAn19991 at 10^6^ PFU and showed self-limiting disease with no clinical signs indicative of CNS infection but did lose weight relative to a control group between 2 and 5 dpi. Inflammation of the spleen was determined based on an increase in the spleen’s relative percentage of body weight, and elevated levels of transaminases in the liver indicate tissue damage was caused by OROV infection. Viral load was detected in the blood (100%), liver (63%) and spleen (100%) of mice challenged with OROV, quantified by both qRT-PCR of the OROV S-segment and by plaque assay, which produced titres between 10^1^ and 10^2^ PFU. A serum antibody response to OROV infection was also confirmed by a plaque reduction neutralisation test (PRNT) [45].

**Table 3:** Overview of OROV immune-competent mouse models.

Publication	Virus		Animals				Study Design				
	Strain	Passage History	Species	Strains	Age	Sex	Challenge Dose	Route of Infection	Inoculation Site	Group *n*	Primary Endpoints
Livonesi et al., 2006 [30]	BeAn19991	Mouse i.c passage	Mouse	SWR/J	Newborn	Mixed	10 LD_50_ *^1^	Intraperitoneal	Abdomen	16	Survival, weight loss, viral load
Santos et al., 2012 [43]	BeAn19991	Mouse i.c passage, HeLa cells	Mouse	BALB/c	Newborn	Mixed	10^6.25^ TCID_50_ *^1^	Subcutaneous	dorsal lumbar	5–6	Survival, weight loss, clinical signs, histopathology, viral load
Santos et al., 2014 [44]	BeAn19991	Mouse i.c passage, HeLa cells	Mouse	BALB/c	3- week-old	Mixed	10^6.25^ TCID_50_ *^1^	Subcutaneous	dorsal lumbar	10	Extensive brain histopathology
Stubbs et al., 2021 [37]	BeAn19991	Vero cells	Mouse	C57BL/6	6- week-old	Male	10^6^ FFU *^1^	Subcutaneous	dorsal lumbar	5	Survival, weight loss, temperature, viral load
Da Silva Menegatto et al., 2023 [45]	BeAn19991	Vero cells	Mouse	BALB/c	3- week-old	Mixed	10^6^ PFU *^1^	Subcutaneous	dewlap	11	Survival, weight loss, histopathology, neutralising antibody titres, ROS markers, viral load

*^1^ Units are as detailed in published work and were not converted due to incompatible assay principles.

### 3.3. Immune-Deficient Mouse Models

An overview of immune-deficient mouse models used in OROV research is shown in Table 4.

IFIT1^-/-^ C57BL/6 mice were used to assess the role of interferon-induced protein with tetratricopeptide repeats 1 (IFIT1) in the OROV immune response. Adult C57BL/6 mice were challenged with a subcutaneous injection of 10^5^ FFU OROV BeAn19991 into the footpad and monitored for three weeks post-challenge. No difference in weight loss, survival or viral load was observed when comparing to wildtype mice challenged with OROV [46].

A 2015 paper by Proenca-Modena et al. modelled OROV infection in range of single and double knockout C57BL/6 mice and identified type-I interferon signalling as a major limiter of OROV pathogenicity, especially the type-I interferon receptor (IFNAR) and interferon regulation factors (IRF3 and IRF7) [47]. Wildtype and knockout C57BL/6 mice, aged 3 weeks old, were challenged, via the subcutaneous route through the footpad, with 10^6^ FFU of OROV BeAn19991. While wildtype mice failed to develop disease, immune-deficient mice with IFNAR, IRF3 and IRF7 or mitochondrial antiviral signalling (MAVS) knockouts presented with severe and lethal disease. No clinical signs indicative of CNS infection were observed, and virus was detected in the CNS tissues of only 2 out of 15 mice at 4 dpi. OROV was detected at high titres in the spleen and liver and was detectable earlier at 2 dpi in IFNAR^-/-^ mice compared to other knockouts where virus was detectable from 4 dpi onwards. Further systemic spread was observed in the immune-deficient models, with virus also detectable at lower titres in blood, kidney and lung tissues [47].

A follow-up study identified the role of IRF5 in limiting neuroinvasive disease in mice, its knockout resulting in more severe disease than the IRF3 and IRF7 knockouts, though the precise mechanism which limits the spread of OROV in the CNS remains unknown [48]. A recent paper highlighted the importance of MyD88 signalling, upstream of IRF5, and B cell maturation in the immune response to OROV infection [49]. Adult IRF5-deficient C57BL/6 mice, which lack mature B cells [50], developed terminal disease whereas T cell-deficient and wildtype C57BL/6 mice showed no clinical signs of OROV infection. MyD88-deficient mice also demonstrated severe disease, likely due to lower serum IgM and IgG levels in the absence of MyD88 signalling [49].

A recombinant OROV expressing a ZsGreen fluorescence gene in place of NSm has also been tested in wildtype and IFNAR^-/-^ mice. The recombinant virus caused similar disease to wildtype OROV strains, despite replicating to approximately 1-log lower when cultured in Vero E6s and A549 cells [51].

A 2021 transmission study in AG129 mice (IFNα/β/ɣ^-/-^ knockout mice) highlights the fact that the mosquito midgut barrier limits the efficient replication and transmission of OROV. Peak viraemia was identified in the mice for use in a transmission model. OROV infected with high lethality, resulting in >80% mortality by 7 dpi, with viral load in the blood peaking at 3–4 dpi. Results showed that OROV was unable to efficiently replicate in *A. aegypti*, *A. albopictus* and *C. quinquefasciatus* after feeding directly on viraemic mice, or when feeding on infectious blood meal containing 10^6^ PFU OROV BeAn19991. Mosquitos were found to be able to transmit OROV to mice only after receiving an injection containing a 10^3^ PFU dose. The possibility of low-efficiency transmission through feeding was considered, with OROV potentially replicating below the limit of detection by RT-qPCR or requiring a higher dose to transmit [52].

The nucleoside analogue 4′-Fluorouridine was assessed in adult IFNα/β^-/-^ BALB/c mice after showing antiviral activity in vitro. Doses ranged from 0.3 mg/kg to 10 mg/kg, administered by oral gavage, before receiving a 30 CCID_50_/mL subcutaneous challenge of OROV BeAn19991 followed by daily dosing to 7 dpi. 4′-Fluorouridine showed a strong protective effect, with 100% survival when mice received doses of 1 mg/kg or higher and 100% mortality in the mock treatment group. Mice treated with any 4′-Fluorouridine showed reduced viral load in blood and tissues when sacrificed at 4 dpi around the expected peak of viraemia. 4′-Fluorouridine was also well tolerated, with weight loss observed only in the high dose group, which eventually recovered [29].

**Table 4:** Overview of OROV immune-deficient mouse models.

Publication	Virus		Animals					Study Design				
	Strain	Passage History	Species	Strains	Knockouts	Age	Sex	Challenge Dose	Route of Infection	Inoculation Site	Group *n*	Primary Endpoints
Pinto et al., 2015 [46]	BeAn19991	Vero cells	Mouse	C57BL/6	IFIT1	6–8-week- old	Female	10^5^ PFU *^1^	Subcutaneous	Footpad	8–12	Survival, viral load, immune- phenotyping
Proenca- Modena et al., 2015 [47]	BeAn19991	Vero cells	Mouse	C57BL/6	IFNAR, IFNβ, MDA5, MAVS, IRF3 and IRF7	5–6-week- old	Mixed	10^6^ FFU *^1^	Subcutaneous	Footpad	23–40	Survival, weight loss, histopathology, liver damage, immune- phenotyping, viral load
Proenca- Modena et al., 2015 [48]	BeAn19991	Vero cells	Mouse	C57BL/6	IFNAR, IRF5, IRF3 and IRF7, IRF3 and IRF5 and IRF7	5–6-week- old	Mixed	10^6^ FFU *^1^	Subcutaneous	Footpad	25–39	Survival, weight loss, histopathology, liver damage, immune- phenotyping, viral load
de Mendonça et al., 2021 [52]	BeAn19991	Vero cells	Mouse	AG129	IFNAR, IFNɣ	3- week- old	Mixed	10^6^ PFU *^1^	Intraperitoneal	Abdomen	15–25	Survival, viral load, transmission confirmed by RT-qPCR
Gunter et al., 2024 [51]	BeAn19991-derived recombinant OROV	Vero, BSR-T7/5, A549	Mouse	C57BL/6 B6(Cg)	IFNAR	6- week- old	Mixed	10^1^ TCID_50_, 10^4^ TCID_50_ *^1^	Subcutaneous	Site not stated	5	Survival, weight loss, histopathology, viral load
Toledo-Teixeira et al., 2025 [49]	BeAn19991	Vero cells	Mouse	C57BL/6	IFNAR, Rag1, CD19-Cre, MyD88	4–6-week-old, 9–12-week-old	Mixed	10^5^ PFU *^1^	Subcutaneous	Footpad	3–5	Survival, weight loss, histopathology, neutralising antibody titres, immune- phenotyping, viral load
Westover et al., 2025 [29]	240023	Vero cells	Mouse	BALB/c	IFNAR	6–8-week- old	Mixed	30 CCID_50_ *^1^	Intraperitoneal	Abdomen	4–5	Survival, weight loss, temperature, viral load

*^1^ Units are as reported in the published manuscripts and were not converted due to incompatible assay principles.

### 3.4. Non-Human Primate Models

An overview of OROV NHP models is shown in Table 5.

Despite their role as a sylvatic cycle host being confirmed since the 1960s [1], NHP models have been neglected for in vivo model for OROV until recently. A 2025 paper by Yee et al. assessed the response of multiple NHP species to infection by OROV, including pigtail and rhesus macaques, as well as vervet and sabeus African green monkeys. Each monkey species received a subcutaneous challenge of either 10^3^, 10^5^ or 10^6.5^ PFU of OROV TRVL 9760. Viraemia presented early at 2 dpi, peaking at 3 to 4 dpi, and persisted to 9 dpi with no dose-dependent effect being observed. Viral replication was observed to be lower in vervet African green monkeys and as such they were considered unsuitable as an NHP model for OROV. No monkeys presented with clinical signs in response to OROV infection regardless of dose, a notable dissimilarity from the presentation of human disease [53]. The current rate of asymptomatic OROV infection in humans is not fully understood [19] and it is estimated that other orthobunyaviruses such as La Crosse cause upwards of 100,000 asymptomatic infections in the USA each year [49]. As such, we cannot currently be certain that the disease presentation of OROV in humans and NHP diverges.

**Table 5:** Overview of OROV NHP models.

Publication	Virus		Animals			Study Design				
	Strain	Passage History	Species	Age	Sex	Challenge Dose	Route of Infection	Inoculation Site	Group *n*	Primary Endpoints
Yee et al., 2025 [53]	TRVL 9760	Vero cells	Pigtail macaques	16–19	Male	10^3^ PFU 10^4^ PFU 10^5^ PFU	Subcutaneous	Site not stated	4–6	Survival, immune phenotyping, neutralising antibody titres, viral load
			Rhesus macaques	9–16	Mixed					
			Sabius African green monkeys	5–14	Male					
			Vervet African green monkeys	unknown	Mixed					

## 4. Discussion

An ideal in vivo model would recapitulate the full course of human disease in animals using a subcutaneous challenge to emulate a natural route of infection, at a dose typically transmitted via bite. The model species would develop a similar pathogenesis and clinical presentation to that seen in humans, including recurrent fever, persistent viral load in fluids, comparable systemic spread and a similar incidence and severity of neurological symptoms. This would create a more suitable test system for safety and efficacy testing of candidate therapeutics and vaccines.

Until recently, no NHP model for OROV had been characterised, despite being a sylvatic host. Although asymptomatic to infection by OROV TRVL 9760, they show a robust immune response that confers protection against secondary infection based on a reduction in viral titres relative to primary infection, though their utility for the assessment of vaccine candidates is limited by the apparent absence of clinical signs of infection [53]. Considering the possibility of high asymptomatic case rates [18,54], further investigation is warranted to better characterise the symptomatology of OROV disease in NHPs. Emerging OROV strains, including those responsible for the first human deaths to OROV, have yet to be tested in NHPs and may prove to be more pathogenic [55]. Additionally, NHP models are also more limited by housing and enrichment costs, as well as requiring stricter ethical constraints, leading to reduced scale.

Neonatal mice also show a rapid and lethal disease progression and are highly susceptible, while adult mice are resistant to OROV infection and do not show signs of disease. As with other similar arboviruses modelled in wildtype mice, such as Zika [56] and Chikungunya [57] viruses, young age and the disruption of type-I interferon signalling enhance the lethality of OROV infection, supporting the notion that the innate interferon response plays a critical role in susceptibility. Similarly, in NHPs, Yee et al. noted the up-regulation of genes associated with the type-I interferon response at peak viraemia [53].

As a consequence of resistance to OROV in adult mice, immune-deficient models have been preferred and have been used to highlight key pathways in the immune response to OROV [48,50]. Mice have also been used for the preliminary assessment of candidate antivirals against OROV, but the efficacy of therapeutics in this model may not be representative of their efficacy in an immune-competent host, limiting the model’s utility for preclinical assessment. Differences between the mouse and human brains also limit its use for investigating the neuroinvasive properties of OROV [28,58].

Humanised mice may present alternative pathogenicity models for OROV, demonstrating a more human-like immune response, and have been established for other arboviruses such as Dengue and Zika [59,60]. A recent paper, in preprint at the time of writing, made use of C57BL/6 mice with a human STAT2 knock-in to better replicate the symptoms of Zika virus infecting human infants by vertical transmission [60]. Another recent preprint has established a vertical transmission model for OROV in mice, showing systemic spread and efficient viral replication in embryonic mice [61]. Considering recent reports of congenital anomalies [28], there is an urgent need for further work to assess the vertical transmission of OROV.

Syrian hamsters show potential as a model for use in pathogenicity and vaccine or antiviral efficacy studies, with low cost and housing requirements [62]. Adult hamsters are naturally susceptible to OROV and present with more obvious clinical signs compared to mouse strains or NHPs [42]. While recurrent fever was not observed in hamsters as in humans, liver damage, persistent viral load, and CNS spread are features of OROV pathology in the hamster model that are similar to what has been described in some human cases [12,21,22,23,42]. As such, Syrian hamsters may be a more suitable model for the initial assessment of vaccine and antiviral candidates.

The majority of small animal models for OROV have only characterised the ancestral BeAn19991, and the closely related TRVL 9760 strain has been tested in NHP studies. Considering that recent outbreaks are responsible for different pathogenesis, including the first fatal cases of Oropouche fever in humans [28], and that these emergent strains are both genotypically and immunologically distinct from BeAn19991 [55], there is an urgent need for them to be studied in vivo. Only one study has assessed the recent clinically relevant 240023 isolate, noting more efficient growth in cell culture compared to BeAn19991, but similar susceptibility in IFNAR^-/-^ mice [29]. Further assessment of clinically relevant isolates should be carried out. Syrian hamsters may be ideal, as they are less rapidly susceptible with a broader clinical presentation than the mouse model.

Three-dimensional organoid models are developing in complexity relative to 2D cultures and are enabling a better understanding of OROV’s neuropathology [28,63], as well as a reduction in the animal burden of OROV research in line with the 3R principles. Almeida et al. demonstrated the utility of organoid models by using adult human brain cultures to identify both the spread of OROV to microglia and neurons and an inflammatory response to infection [28]. As these systems cannot yet effectively simulate organ–organ interactions or be used to characterise broader systemic disease, the development of, and subsequent recovery from, clinical signs of disease in animal model systems remains necessary for the robust evaluation of therapeutics [64].

Three reassortants have already been identified to our knowledge [18,19,20], with more recent clinical isolates that have caused the first known human fatalities showing increased variation in L segment regions encoding the RNA polymerase and M segment regions encoding the Gc surface protein [65]. Given the potential for increased disease severity based on recent case reports and the increasing divergence from typically used laboratory strains, there is a need to confirm the symptomatology of OROV reassortants and recent clinical isolates in vivo to decipher their importance and assess the likelihood of risk of increased disease.

With the development of new antivirals and vaccines against OROV [29,36], standardised preclinical models will form a pivotal role in their evaluation. Further work to develop these models is therefore crucial to aid in identifying suitable and effective countermeasures and to further our understanding of the kinetics involved in disease pathogenesis.

In summary, the hamster model provides a representation of systemic infection, mirroring several severe symptoms observed in human case reports, such as liver damage, viraemia and neurological signs. While useful, the outbred nature of the hamster model introduces variability, and limited reagent availability hinders a more comprehensive immunological assessment. Key endpoints for this model include survival, weight loss, possible fever spikes, clinical signs of febrile and neurological disease and the quantification of viral load in fluids and liver and CNS tissues. Wild-type mice are resistant to OROV, necessitating the use of immune-deficient mice for susceptibility. IFNAR^-/-^ mice have been used to investigate specific pathways in the immune response. As such, this model may not reflect a human immune response. Endpoints in the mouse model usually include survival, weight loss, quantification of viral load and a robust immunological assessment. The NHP model has an asymptomatic presentation following OROV infection but demonstrates a robust immune response. As such, it is unviable as a pathogenicity model. The NHP’s similarity to the human immune system makes it valuable for safety and efficacy testing of vaccines and antiviral countermeasures. Endpoints for the NHP model include quantification of viral load and in-depth immunological assessment.

## Data Availability

No new data were created or analysed in this study. Data sharing is not applicable to this article.

## Abbreviations

The following abbreviations are used in this manuscript:

CD	Cluster of Differentiation
CNS	Central Nervous System
Da	Dalton
dpi	Days Post-Infection
ds	Double-Stranded
FFU	Foci Forming Unit
IFNAR	Interferon Alpha Receptor
IFN	Interferon
Ig	Immunoglobulin
IHC	Immunohistochemistry
IRF	Interferon Regulatory Factor
IQTV	Iquitos Virus
LD50	50% Lethal Dose
MDDV	Madre de Dios Virus
MyD	Myeloid Differentiation
NHP	Non-Human Primate
OROV	Oropouche Virus
PCR	Polymerase Chain Reaction
PERDV	Perdões Virus
PFU	Plaque Forming Unit
PRNT	Plaque-Reduction Neutralisation Test
q	Quantitative
Rag	Recombination Activating Gene
RdRp	RNA-Dependent RNA-Polymerase
RNA	Ribonucleic Acid
RT	Reverse Transcription
SARS-CoV-2	Severe Acute Respiratory Syndrome
SMLD50	50% Suckling Mouse Lethal Dose
TCID50	50% Tissue Culture Infectious Dose
UTR	Untranslated Region
Wt	Wildtype
